# Molecular mechanisms of nutlin-3 involve acetylation of p53, histones and heat shock proteins in acute myeloid leukemia

**DOI:** 10.1186/1476-4598-13-116

**Published:** 2014-05-21

**Authors:** Ingvild Haaland, Jill A Opsahl, Frode S Berven, Håkon Reikvam, Hanne K Fredly, Ragnhild Haugse, Bernd Thiede, Emmet McCormack, Sonia Lain, Øystein Bruserud, Bjørn Tore Gjertsen

**Affiliations:** 1 Department of Clinical Science, Hematology Section, University of Bergen, Bergen N-5021, Norway; 2 Department of Biomedicine, Proteomics Unit at University of Bergen (PROBE), University of Bergen, Bergen N-5021, Norway; 3 The Biotechnology Centre of Oslo, University of Oslo, Oslo N-0317, Norway; 4 Department of Internal Medicine, Haukeland University Hospital, Bergen N-5021, Norway; 5 Department of Microbiology, Tumor and Cell Biology, Karolinska Institute, Stockholm SE-171777, Sweden; 6 Centre for Cancer Biomarkers, Department of Clinical Science, University of Bergen, Bergen N-5021, Norway

**Keywords:** Nutlin-3, Acetylation, SILAC, p53, Histone, Heat shock protein, Acute myeloid leukemia

## Abstract

**Background:**

The small-molecule MDM2 antagonist nutlin-3 has proved to be an effective p53 activating therapeutic compound in several preclinical cancer models, including acute myeloid leukemia (AML). We and others have previously reported a vigorous acetylation of the p53 protein by nutlin-treatment. In this study we aimed to investigate the functional role of this p53 acetylation in nutlin-sensitivity, and further to explore if nutlin-induced protein acetylation in general could indicate novel targets for the enhancement of nutlin-based therapy.

**Results:**

Nutlin-3 was found to enhance the acetylation of p53 in the human AML cell line MOLM-13 (wild type TP53) and in TP53 null cells transfected with wild type p53 cDNA. Stable isotope labeling with amino acids in cell culture (SILAC) in combination with immunoprecipitation using an anti-acetyl-lysine antibody and mass spectrometry analysis identified increased levels of acetylated Histone H2B, Hsp27 and Hsp90 in MOLM-13 cells after nutlin-treatment, accompanied by downregulation of total levels of Hsp27 and Hsp90. Intracellular levels of heat shock proteins Hsp27, Hsp40, Hsp60, Hsp70 and Hsp90α were correlated to nutlin-sensitivity for primary AML cells (*n* = 40), and AML patient samples with low sensitivity to nutlin-3 tended to express higher levels of heat shock proteins than more responsive samples. Combination therapy of nutlin-3 and Hsp90 inhibitor geldanamycin demonstrated synergistic induction of apoptosis in AML cell lines and primary AML cells. Finally, TP53 null cells transfected with a p53 acetylation defective mutant demonstrated decreased heat shock protein acetylation and sensitivity to nutlin-3 compared to wild type p53 expressing cells.

**Conclusions:**

Altogether, our results demonstrate that nutlin-3 induces acetylation of p53, histones and heat shock proteins, and indicate that p53 acetylation status and the levels of heat shock proteins may participate in modulation of nutlin-3 sensitivity in AML.

## Background

Acute myeloid leukemia (AML) is a quickly progressive malignant disease of the myeloid lineage of hematopoietic cells, where overall three-year survival is below 20% for patients above 65 years [1]. As elderly patients do not tolerate the intensive chemotherapy and stem cell transplantation of current treatment regimes [2], the development of less toxic and more specific targeted therapy is necessary. Small-molecule MDM2 inhibitors like nutlin-3 have emerged as a potent and promising treatment option for cancers harboring wild type TP53, including AML [3-5], and the oral formulation of nutlin-3, RG7112, has completed the first early phase clinical trials for both solid cancers and hematological malignancies [6-8]. Intriguingly, these small-molecule p53 activators have demonstrated selective toxicity for cancer cells versus normal cells [4,9], and may also induce reversible cell cycle arrest of normal cells to protect them from adverse effects of conventional chemotherapy [10].

While nutlin-3 initially was thought to exert its anti-cancer activity specifically through inhibition of the p53-MDM2 interaction, recent studies have demonstrated dual-targeting and p53 independent effects of nutlin-3 [11-13]. The efficacy of nutlin-3 and other MDM2 inhibitors in hematological malignancies seems however largely to depend on the expression and activation of wild type p53 [4,9,14,15]. In addition to TP53 mutational status, several other molecular mechanisms have been shown to affect the sensitivity to MDM2 targeted therapy, including FLT3 and NPM1 mutational status [15-17], E2F-1 transcriptional activity [18], overexpression of MDMX [19], and MDM2 levels [4,9]. The observed resistance to nutlin-3 in cohorts of AML patients could be explained by the extensive heterogeneity and range of molecular abnormalities of the disease [2,4]. For instance, aberrant recruitment of histone deacetylases (HDACs) and overexpression of heat shock proteins (Hsps) have been shown to be involved in the molecular pathogenesis and therapy response of AML [20,21], and could therefore be considered as potential therapeutic targets to combine with MDM2 inhibition. Inhibitors of HDACs and Hsp90 have been found to enhance p53 acetylation and inhibit MDMX, and synergize with nutlin-3 to induce p53-mediated apoptosis [22-24]. The direct effect of nutlin-3 on regulation of histones and heat shock proteins has however not been determined.

In this study, we aimed to investigate mechanisms underlying the anti-leukemic activity of nutlin-3. We examined the functional role of p53 acetylation in nutlin-sensitivity, and hypothesized that nutlin-induced acetylation of other proteins than p53 would be of importance for the anti-leukemic effect of nutlin-3. Combining immunoprecipitation of acetylated proteins with quantitative proteomics, we identified novel targets of nutlin-induced acetylation, and investigated their participation in the nutlin-mediated response in AML cell lines and primary AML cells.

## Results

### Nutlin-3 enhances p53 acetylation independently of total levels of p53

**Figure 1:**
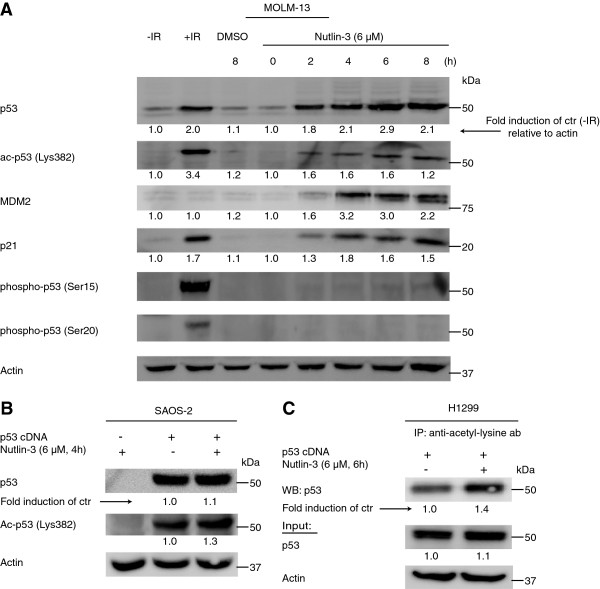
**Nutlin-induced acetylation of p53. (A)** MOLM-13 cells were treated with 6 μM nutlin-3 for 0, 2, 4, 6 and 8 hours. Cells subjected to irradiation (IR) (25 Gy, 2 hours incubation) were included as a positive control, while cells treated with DMSO for 8 hours was used as a negative control. Western blotting was performed using antibodies against p53, acetylated p53 (Lys382), phoshorylated p53 (Ser15), phosphorylated p53 (Ser20), MDM2 and p21. Actin was used as loading control. **(B, C)** SAOS-2 and H1299 cells were transiently transfected with cDNA of p53 and treated with DMSO (control) or 6 μM nutlin-3 for 4 or 6 hours. For SAOS-2 cells, Western blotting was performed using antibodies against p53, acetylated p53 (Lys382) and actin. For H1299 cells, immunoprecipitation was performed using an anti-acetyl-lysine antibody, followed by Western blots of p53. Also Western blots of p53 and actin from total lysate used in the immunoprecipitation are shown. Bands were quantified using region of interest imaging analysis, and values are given as fold induction of control relative to actin.

While nutlin-3 previously has been shown to enhance the acetylation of p53 [22,23], it is not clear whether this is only a consequence of the increase in total levels of p53. The human AML cell line MOLM-13 (TP53 wild type) treated with nutlin-3 at increasing time points demonstrated increased levels of p53, MDM2, p21 and acetylated p53 (Lys382), while the induction of phosphorylated p53 (Ser15 and Ser20) was diminishable (Figure 1A). To investigate if nutlin-3 could induce acetylation of p53 independent of a substantial increase in total p53, we transfected the human osteosarcoma cell line SAOS-2 (TP53 null) with a cDNA construct of p53 and treated the cells with nutlin-3. The results demonstrated a higher increase in acetylated p53 (Lys382) compared to total levels of p53 after nutlin-treatment (Figure 1B). Similarly, the human lung cancer cell line H1299 (TP53 null) transfected with p53 and treated with nutlin-3, followed by immunoprecipitation with an anti-acetyl-lysine antibody, demonstrated a high increase in levels of acetylated p53 after nutlin-treatment, and only a small increase in total levels of p53 (Figure 1C).

### Nutlin-3 enhances the acetylation of histone H2B and heat shock proteins Hsp27 and Hsp90

**Figure 2:**
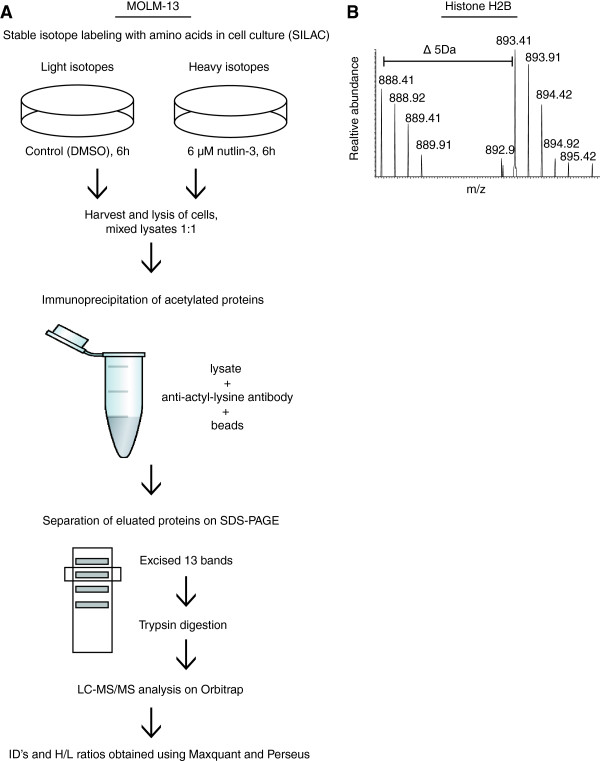
**Nutlin-induced modulation of acetylated proteins in the AML cell line MOLM-13. (A)** MOLM-13 cells were subjected to stable isotope labeling with amino acids in cell culture (SILAC). Cells were labeled with either light (L-Lysine-2HCl, L-Arginine-HCl) or heavy (^13^C_6_ L-Lysine-2HCl, ^13^C_6_^15^ N_4_ L-Arginine-HCl) isotopes of amino acids and treated with DMSO (control) or 6 μM nutlin-3, respectively, for 6 hours. Cells were harvested and lysed, and lysates were mixed at a ratio of 1:1 (5 mg protein of each). The lysate was precleared with uMACs protein G Microbeads, then precleared with beads and an unspecific antibody (rabbit IgG), before immunoprecipitation of acetylated proteins using an anti-acetyl-lysine antibody and Microbeads. Proteins were eluted in 95°C SDS loading buffer and subjected to one-dimensional gel electrophoresis (SDS-PAGE) and staining with Coomassie Blue. Bands were excised and peptides generated by trypsination. Peptides were separated and fragmented using LC-MS/MS (LC-LTQ-Orbitrap) and protein ID’s and H/L ratios were obtained using MaxQuant and Perseus software. **(B)** Representative MS/MS spectrum of peptides derived from Histone H2B.

Based on the critical role of acetylation in nutlin-induced p53 activation, we wanted to examine if nutlin-3 could enhance the acetylation of other proteins than p53. We used stable isotope labeling with amino acids in cell culture (SILAC) [25] in combination with immunoprecipitation of acetylated proteins and mass spectrometry analysis to determine alterations in acetylated proteins after nutlin-treatment in MOLM-13 cells (for work flow and experimental details, see Figure 2A). Only proteins with two ore more peptides that were either two-fold up- or downregulated were considered significant. From 141 proteins identified with two or more peptides, 6 proteins were significantly downregulated and 3 proteins were significantly upregulated in response to nutlin-3 (see Additional file 1: Table S1 text; Additional file 2: Table S1). MDM2 is involved in the regulation of different acetyltransferases and histone deacetylases, and may interact with and promote ubiquitination and deacetylation of other proteins than p53 [26,27]. As nutlin-3 may inhibit interactions between MDM2 and other proteins than p53 [11,28], we hypothesized that nutlin-induced disruption between MDM2 and various proteins would prevent their ubiquitination and promote their acetylation. Hence, we chose to limit our study to acetylated proteins that were upregulated in response to nutlin-treatment.

**Figure 3:**
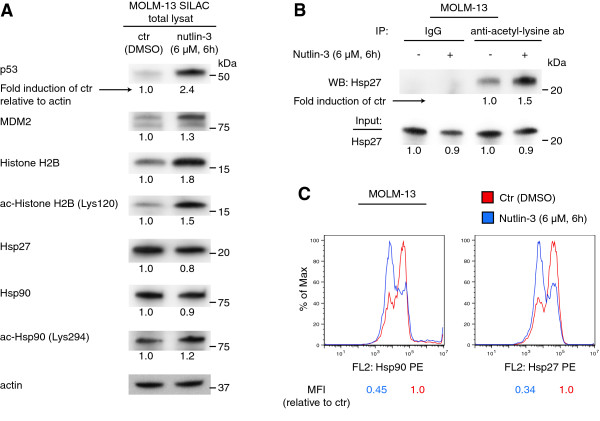
**Validation of nutlin-induced modulation of acetylated proteins in the AML cell line MOLM-13. (A)** Western blots of total lysates from SILAC (stable labeling with amino acids in cell culture) labeled MOLM-13 cells treated with DMSO or 6 μM nutlin-3 for 6 hours using antibodies against p53, MDM2, Histone H2B, acetylated Histone H2B (Lys120), Hsp27, Hsp90, acetylated Hsp90 (Lys294) and actin. Bands were quantified using region of interest imaging analysis, and values are given as fold induction of control relative to actin. **(B)** MOLM-13 cells were treated nutlin-3 as described above, and immunoprecipitations using anti-acetyl lysine antibody or an unspecific antibody (rabbit IgG) were performed. Western blots of Hsp27 of the immunoprecipitated proteins and of the total lysates used in immunoprecipitations are shown. **(C)** Total levels of Hsp90 and Hsp27 in MOLM-13 cells treated with nutlin-3 as described above analyzed by flow cytometry. Results are given as representative flow diagrams and median fluorescence intensity (MFI) relative to control.

Histone H2B and Hsp27 were among the acetylated proteins that were more than two-fold up regulated by nutlin-3 (Table 1) (for example of spectrum, see Figure 2B). While MDM2 has been shown to mediate ubiquitination and deacetylation of histones, leading to transcriptional repression [27], heat shock proteins like Hsp27 and Hsp90 may interact with both MDM2 and p53, and promote MDM2 mediated ubiquitination of p53 [29-31]. Thus, we found it interesting that nutlin-3 could have an effect on regulation of these proteins, and they were selected for validation in Western blots and further analysis. Western blots of total lysates from the SILAC experiment demonstrated upregulation of p53, MDM2, Histone H2B, acetylated Histone H2B (Lys120) and acetylated Hsp90 (Lys294), and downregulation of total levels of Hsp27 and Hsp90 after nutlin-treatment (Figure 3A). Increased levels of acetylated Hsp27 and down regulation of total levels of Hsp27 were validated by immunoprecipitation with an anti-acetyl-lysine antibody in MOLM-13 treated with nutlin-3 (Figure 3B). Decreased total levels of Hsp27 and Hsp90 after nutlin-treatment were further validated by flow cytometry (Figure 3C).

**Table 1:** Acetylated proteins more than two-fold up regulated by nutlin-3 detected by SILAC-based mass spectrometry

**Accession number**	**Protein IDs**	**Name**	**Number of peptides**	**Sequence Coverage, %**	**Ratio of H/L, normalized**
P04792	IPI00025512	Hsp27/HSPB1	2	12.7	2.0741
B4DR52	IPI00646240	Histone H2B	2	14.5	2.3519
Q13765	IPI00797126	Alpha-NAC	4	5.9	3.4854

*Abbreviations: Alpha-NAC* Nascent polypeptide-associated complex subunit alpha, *Hsp27* Heat shock protein 27, *HSBP1* Heat shock protein beta-1, *H/L* ratio between proteins abundance in cells labeled with heavy isotopes compared to cells labeled with light isotopes, *SILAC* stable isotope labeling with amino acids in cell culture.

### Intracellular levels of heat shock proteins and sensitivity to nutlin-3 in primary AML cells

**Figure 4:**
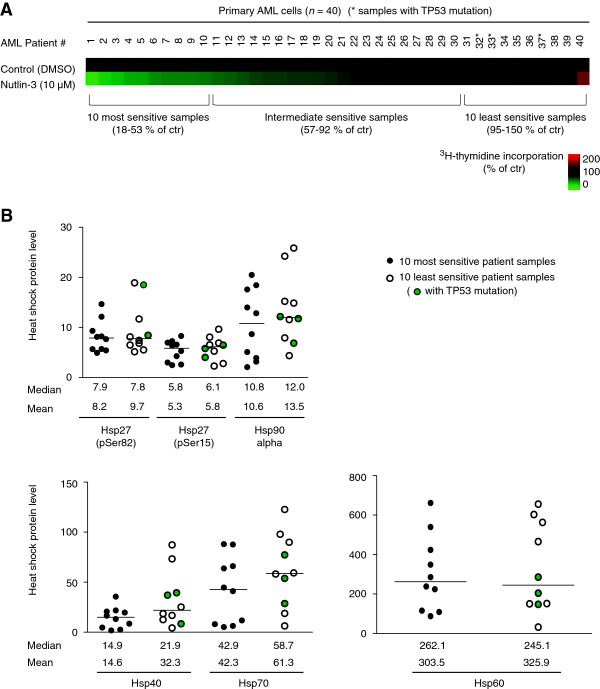
**Intracellular heat shock protein levels and sensitivity to nutlin-3 in primary AML cells. (A)** Sensitivity to nutlin-3 (10 μM, 24 hours) in 40 primary AML samples was determined by ^3^H-thymidine incorporation assay, and samples were analyzed in triplicates. Samples with TP53 mutations are marked with *. Intracellular levels of heat shock proteins Hsp27 (phospho-Ser82), Hsp27 (phospho-Ser15), Hsp40, Hsp60, Hsp70 and Hsp90α for all samples were determined using Hsp/Chaperone 8-plex MultiBead kit and flow cytrometric analysis. Samples were analyzed in duplicates. **(B)** Median values of heat shock protein levels were determined for the patient samples that were sensitive (10 most sensitive ranging from 18-53% viability of control) and non-sensitive (10 least sensitive ranging from 95-above 100% viability of control) to nutlin-3, and are shown in the figure together with values for individual patient samples. Samples with TP53 mutations are indicated. Mean values are given below. For Hsp27 (phospho-Ser82) and Hsp27 (phospho-Ser15) levels are given as u/ml; for Hsp40, Hsp60, Hsp70 and Hsp90α levels are given as ng/ml.

To investigate if levels of different heat shock proteins could affect sensitivity to nutlin-3, intracellular protein levels of Hsp27 (phospho-Ser82), Hsp27 (phospho-Ser15), Hsp40, Hsp60, Hsp70 and Hsp90α were quantified in primary AML cells (*n* = 40) using an Hsp/Chaperone 8-plex MultiBead kit and flow cytometric analysis, while sensitivity to nutlin-3 was determined in ^3^H-thymidine incorporation assay (Figure 4A) (for AML patient characteristics, see Additional file 3). Pearson correlation analysis between nutlin-sensitivity and levels of the different heat shock proteins revealed no significant correlations (data not shown). However, when patient samples were divided into the 10 most sensitive (0 - 53% viability of control) and 10 least sensitive (95 - above 100% viability of control) to nutlin-3, the least sensitive patient samples showed a trend towards higher expression levels of most heat shock proteins, although the differences in median values were not significant (median values for sensitive versus non-sensitive samples for Hsp27(pSer82): 7.9/7.8 u/ml; Hsp27(pSer15): 5.8/6.1 u/ml; Hsp90 alpha: 10.8/12.0 ng/ml; Hsp40: 14.6/21.9 ng/ml; Hsp70: 42.9/58.7 ng/ml; Hsp60: 262.1/245.1 ng/ml) (Figure 4B) (for AML patient characteristics, see Additional file 3). Mean values demonstrated similar results, but with a stronger tendency to elevated levels of all heat shock proteins in non-sensitive samples, although differences were not statistically significant (Figure 4B). In this data set, patient samples with both wild type and mutated TP53 were included. Given the fact that samples with mutated TP53 could respond differently to nutlin-3 than those with wild type TP53, we also performed analyses on the patient set including only patient samples with confirmed wild type TP53 (*n* = 31). Also for this set of samples, there were no significant correlations between nutlin-sensitivity and levels of the different heat shock proteins, but a tendency to elevated levels of all heat shock proteins (both median and mean values) in the least sensitive samples, although there were no significant differences for the 10 most sensitive versus the 10 least sensitive for this patient set either (Figure S1) [Additional file 4].

### Inhibition of Hsp90 sensitizes AML cells to nutlin-induced apoptosis

**Figure 5:**
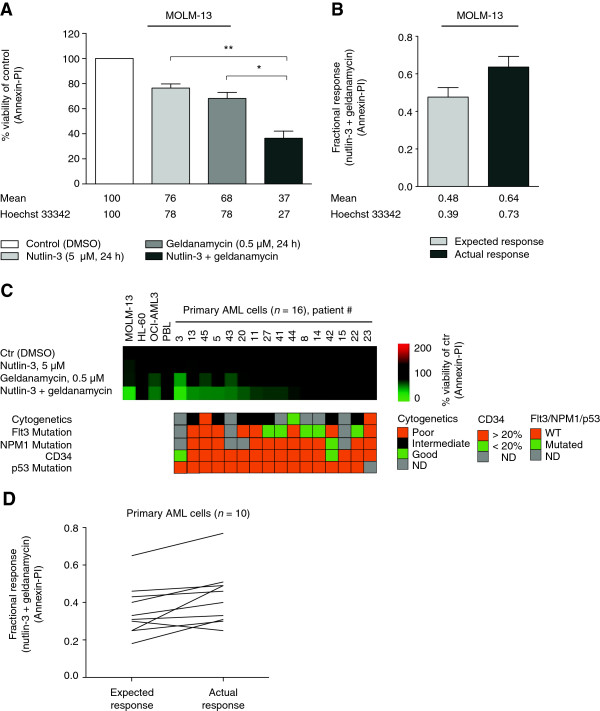
**Combinational therapy of nutlin-3 and geldanamycin in AML cells lines and primary AML cells. (A)** MOLM-13 cells were treated with DMSO (control), nutlin-3 (5 μM), geldanamycin (0.5 μM) or the combination of nutlin-3 and geldanamycin for 24 hours and analyzed by Annexin-PI viability assay. Reduction of viability by the combinational treatment was compared to either compound alone (**p < 0.01, *p < 0.05). Results are from three independent experiments and error bars represent standard error of mean. Viability values determined by staining with Hoechst 33342 are given below the Annexin-PI data. **(B)** Synergism calculated by Bliss Independence analysis for results from Figure (A) showing expected and actual response for the combinational treatment **(C)** Sensitivity to nutlin-3 (5 μM), geldanamycin (0.5 μM), or the combination of both for 24 hours in AML cell lines MOLM-13, HL60, OCI-AML3, normal peripheral blood lymphocytes and 16 primary AML samples analyzed by Annexin-PI and visualized using TMEV microarray software, with corresponding clinical parameters for each of the patients. **(D)** Bliss Independence analysis of expected and actual response for the combinational therapy for each of the individual responding AML patient samples analyzed by Annexin-PI (*n* = 10).

As nutlin-3 was found to acetylate and inhibit heat shock proteins, we investigated their functional role in nutlin-sensitivity. Hsp90 plays a central role in leukemogenesis, and preclinical and preliminary clinical data indicate beneficial effects of Hsp90 inhibitors in the treatment of AML [21,32]. Moreover, both nutlin-3 and hsp90 inhibitors are shown to activate p53 [33], and inhibition of Hsp90 has been shown to antagonize MDMX and synergize with nutlin-3 to induce p53-mediated apoptosis in solid tumors [24]. Hence, we used the Hsp90 inhibitor geldanamycin to determine if Hsp90 inhibition could enhance the anti-leukemic effect of nutlin-3. MOLM-13 cells treated with nutlin-3, geldanamycin or the combination of both, demonstrated increased sensitivity to the combination therapy compared to either agent alone determined by Annexin-PI viability assay (**p < 0.01, *p < 0.05) or staining with Hoechst 33342 (Figure 5A). Synergism for the interaction of nutlin-3 and geldanamycin was calculated using Bliss independence analysis, in which the fractional response of a combination of two drugs equals the sum of the two fractional responses minus their product. From the response to each of the drugs alone, the expected response to the combination was calculated. If there was a positive difference between the actual and expected response, the combination was considered synergistic [34]. Bliss Independence analysis of the data revealed synergistic apoptosis induction with a higher actual response than expected response for the combinational therapy for both assays (Figure 5B). The combinational therapy was also tested in the AML cell lines OCI-AML3 (wild type TP53, wild type FLT3) and HL60 (deleted TP53), and in normal peripheral blood lymphocytes, demonstrating decreased sensitivity in cells with wild type TP53 and wild type FLT3 compared to cells with wild type TP53 and mutated FLT3, and no effect in cells with deleted TP53 or in normal cells in Annexin-PI viability assay (Figure 5C). Primary AML cells from 16 patients demonstrated various sensitivity to the combinational treatment in Annexin-PI viability assay (Figure 5C); 10 out of 16 patients responded to the treatment, and 9 out of the 10 responsive patient samples demonstrated synergism, with a higher actual response than expected response for the combinational treatment (Figure 5D) (for AML patient characteristics, see Additional file 3).

### Role of p53 acetylation in nutlin-sensitivity and regulation of heat shock proteins

**Figure 6:**
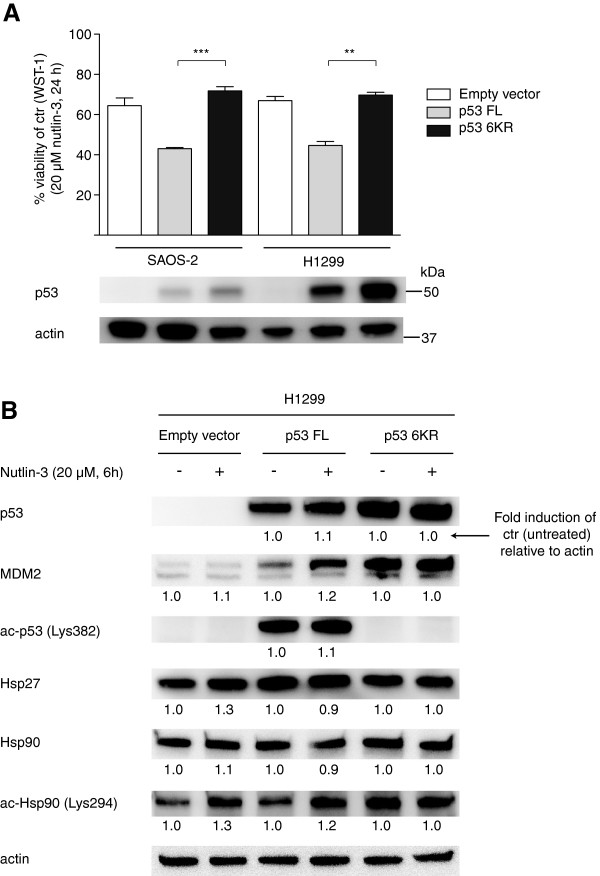
**Functional role of p53 acetylation in nutlin-sensitivity. (A)** SAOS-2 and H1299 cells were transiently transfected with empty vector, p53 full length (FL) or the actylation defective mutant p53 6KR and treated with 20 μM nutlin-3 for 24 hours. Cell viability was determined using the WST-1 viability/proliferation assay (***p < 0.001, **p < 0.01). Results were analyzed in triplicates in tree independent experiments and error bars represent standard error of mean. Transfections were verified in Western blots with antibodies against p53 and actin. **(B)** H1299 cells were transiently transfected with empty vector (EV), p53 full length (FL), or p53 6KR and treated with DMSO or 20 μM nutlin-3 for 6 hours. Western blotting was performed using antibodies against p53, MDM2, acetylated p53 (Lys382), Hsp27, Hsp90 and acetylated Hsp90 (Lys294) and actin. Bands were quantified using region of interest imaging analysis, and values are given as fold induction of control (DMSO treated sample for each transfection for EV/p53 FL/p53 6KR) relative to actin.

In order to examine the functional role of p53 acetylation in nutlin-sensitivity, we transfected SAOS-2 and H1299 cells with constructs of p53 full length (p53 FL) and an acetylation defective mutant (p53 6KR; simultaneous mutation of lysine residues 370, 372, 373, 381, 382, and 386 to arginine residues) [35]. Nutlin-treatment demonstrated reduced sensitivity to nutlin-3 in cells transfected with p53 6KR compared to cells transfected with p53 FL in WST-1 viability/proliferations assay for both cell lines (***p < 0.001, **p < 0.01) (Figure 6A). To investigate the role of p53 and p53 acetylation in nutlin-induced modulation of heat shock proteins, we transfected H1299 cells with empty vector, p53 FL and p53 6KR as described above and treated the cells with nutlin-3, followed by Western blot analysis of p53, MDM2, acetylated p53 (Lys382), Hsp27, Hsp90 and acetylated Hsp90 (Lys294) (Figure 6B). The results demonstrated nutlin-induced increase of Hsp27, Hsp90 and acetylated Hsp90 in cells without p53, downregulation of Hsp27 and Hsp90 and upregulation of acetylated Hsp90 in cells with wild type p53, and no changes in heat shock protein regulation in response to nutlin-3 in cells transfected with p53 6KR. The transfection of p53 6KR itself did however cause an increase in levels of acetylated Hsp90 compared to cells transfected with empty vector. There was no difference in cell viability by the transfection itself between empty vector and p53 6KR, while cells transfected with p53 FL demonstrated a small increase in cell viability compared to empty vector and p53 6KR (data not shown).

## Discussion

Small-molecule MDM2 antagonists like nutlin-3 have demonstrated beneficial effects in cellular and preclinical models of various cancer types, including AML [5]. This type of non-genotoxic specific targeted therapy holds promise for the treatment of AML patient groups lacking satisfactory treatment options due to toxicity and complications associated with current treatment regimes [2]. A better understanding of the molecular mechanisms behind the anti-cancer activity of these compounds is however needed for further development of this type of therapy. The identification of molecular targets that could affect the sensitivity to the drug may be of importance for classification of patient groups that would benefit from the therapy, and for designing combinational therapy in order to overcome resistance, lower doses, and reduce side effects.

It is well established that expression and activation of p53 is a major determinant in nutlin-induced apoptosis [3,4,9]. Previous studies have also shown that nutlin-3 enhances the acetylation of p53 in different human cancer cell lines [22,23]. Our results confirm the universality of nutlin-induced p53 acetylation in both AML cell lines and other human cancer cell lines, and furthermore demonstrate that the increase in p53 acetylation is independent of a simultaneous increase in total p53. The experiments applying a p53 acetylation defective mutant clearly illustrate that in addition to expression of p53, the modulation status of p53 is of great importance in nutlin-sensitivity. However, it should be taken into consideration that this mutant also is resistant to MDM2 mediated ubiquitination, resulting in higher expression levels of this mutant compared to wild type p53. Importantly, the p53 6KR mutant shows intact p53 transcriptional activity, but without the inhibitory regulation of MDM2 [35]. Acetylation of p53 has been shown to be essential for its activation and regulation of different processes [36-38], and to play an important role in therapy response [39,40]. Meanwhile, high expression level of p53 is associated with poor prognosis and resistance to therapy in AML [41]. The possibility that the high levels of p53 is a consequence of modifications like acetylation, and that also p53 acetylation status in primary AML samples could provide information about nutlin-sensitivity need to be examined in future experiments. There are several possible explanations regarding the molecular mechanisms behind nutlin-induced p53 acetylation; Disruption of MDM2-p53 interaction could prevent MDM2 mediated ubiquitination or deacetylation of p53 [42,43], or nutlin-3 could prevent MDM2 from interacting with and inhibiting acetyl transferases important for p53 acetylation and activity [44,45]. These and other possible molecular mechanisms need to be further explored.

In general, protein lysine acetylation has been shown to play an important role in regulation of cellular function and cancer cell signaling, also in AML [46,47]. In addition to inhibiting MDM2-p53 interaction and modulating p53, nutlin-3 may affect several other proteins, either as a consequence of p53 transcription-dependent or -independent effects [48], changed interactions between MDM2 and other proteins than p53 [11,28], or direct effect of nutlin-3 interaction with other proteins than MDM2 [12]. Accordingly, we wanted to examine if nutlin-3 could enhance the acetylation of other proteins than p53. The methodology using SILAC in combination with an anti-acetyl-lysine antibody and mass spectrometry analysis has previously successfully been applied to identify and quantify alterations in acetylated proteins in cells treated with HDAC inhibitors, and both histones and heat shock proteins were identified as lysine acetylated [49,50]. The novel observation that nutlin-3 enhances the acetylation of histones, could add information regarding the molecular mechanisms behind the synergism of nutlin-3 and HDAC inhibitors [22,23]. While acetylation of histones is important for their transcriptional activity [20], acetylation of heat shock proteins have been shown to inhibit their chaperone activity and promote their export and extracellular location [51,52]. This could explain the decrease in total levels of Hsp27 and Hsp90 as a consequence of nutlin-induced acetylation of these proteins. The combination of HDAC and Hsp90 inhibitors has demonstrated synergism in leukemia, but antagonism in other tumor models [53]. Also the combination of HDAC inhibitors and nutlin-3 has shown contradictory results in different experimental settings [22,23,54,55]. As for p53, there are several possible mechanisms behind nutlin-induced acetylation of histones and heat shock proteins, including alterations in interaction between MDM2, histones and heat shock proteins or between MDM2 and components involved in regulating the acetylation of these proteins; further investigations are therefore warranted.

p53 and p53 acetylation seemed to be of importance for nutlin-mediated regulation of total and acetylated levels of heat shock proteins (Figure 6B). Nutlin-induced acetylation of Hsp90 occurred also in cells without p53, while downregulation of total levels of Hsp90 and Hsp27 was dependent of wild type p53. Previous studies using another MDM2 inhibitor have also shown downregulation of other heat shock proteins in wild type p53 cancer cells in response to treatment [56]. Cells transfected with a p53 acetylation defective mutant demonstrated increased levels of MDM2 and acetylated Hsp90 by the transfection itself, but no effects on regulation of total or acetylated heat shock proteins in response to nutlin-treatment. In future perspectives, it would be interesting to perform similar experiments with acetylation defective heat shock protein mutants to investigate the role of heat shock protein acetylation in nutlin-induced p53 acetylation.

Sensitivity to both MDM2 and Hsp90 inhibitors is influenced by different molecular mechanisms in AML [15,17]. As high expression of heat shock proteins has been associated with poor prognosis and therapy resistance in AML [21,57], and different heat shock proteins may interact with and inhibit p53 [29,31], we wanted to examine if total levels of different heat shock proteins in AML patient samples could affect the sensitivity to nutlin-3. We did not find any significant correlations between nutlin-sensitivity and concentration of intracellular levels of different heat shock proteins in 40 primary AML samples. However, when the sample cohort was divided into sensitive and non-sensitive patient samples, there was a trend towards higher expression of heat shock proteins in the least sensitive patient samples, although the differences were not significant. Considering the fact that samples with TP53 mutations may respond differently to nutlin-3 compared samples with wild type p53, we also included analyses on the patient set including only samples with wild type TP53 (*n* = 31), with similar results. The number of patient samples is however relatively low; a larger number of patient samples should therefore be included to determine if there are significant differences in heat shock protein levels in nutlin-sensitive versus non-sensitive samples. It would also be of interest to correlate levels of acetylated heat shock proteins and levels of induction of acetylated heat shock proteins in response to nutlin-3 with nutlin-sensitivity in primary AML samples.

To examine the functional effect of heat shock protein inhibition on nutlin-sensitivity, we chose to combine nutlin-3 with the Hsp90 inhibitor geldanamycin. The combination of nutlin-3 with Hsp90 inhibitors has previously demonstrated synergism in solid tumors [24], while nutlin-3 and geldamamycin exhibited various effects in classical Hodgkin’s lymphoma depending on TP53 mutational status [58]. Determination of drug interaction by Bliss independence analysis assumes that the two drugs act through independent mechanisms [34]; nutlin-3 acts as an MDM2 inhibitor, and geldanamycin binds to and inhibits Hsp90 (although the may converge on the same pathway and indirectly affect the same down stream targets). Based on Bliss independence analysis with observed higher actual than expected response for both MOLM-13 cells and 9 out of 10 responsive primary AML samples, we propose that nutlin-3 and geldanamycin would kill cells independently of each other in a synergistic manner. Possible mechanisms may include enhanced Hsp90 inhibition and p53 activation [33]. As Hsp90 has a wide range of client proteins, additional molecular mechanisms behind the observed synergism behind nutlin-3 and Hsp90 inhibitors have been proposed [24]. To eliminate potential off-target effects of geldanamycin, the use of short hairpin RNAs (shRNAs) for stable and specific knockdown of Hsp90 in combination with nutlin-3 could be an option in future experiments. Inhibition of Hsp90 has been shown to induce Hsp27, possibly contributing to antagonizing the anticancer activity of Hsp90 inhibitors [53]. In contrast, inhibition or knock down of Hsp27 also inhibits Hsp90 [59]. Hence, in future studies, it would be interesting to combine nutlin-3 with shRNAs or small molecule oligonucleotides against Hsp27.

In our proteomics approach, we restricted the study to alterations in the lysine acetylome in the whole cell lysate compared to more extensive analysis of the proteome. As no other isolations or fractionations into for example nuclear and cytoplasmatic fractions were performed, a limitation of this procedure may be that only the most abundant proteins were detected. Further investigations could therefore include studying nutlin-induced acetylation and modulation of other less abundant proteins as well.

## Conclusions

In conclusion, our results indicate that acetylation of p53, histones and heat shock proteins may be a part of the molecular mechanisms behind the anti-leukemic activity of nutlin-3. Regulation and function of histones and heat shock proteins in nutlin-sensitivity need to be evaluated in a larger number of primary AML cells, as well as in preclinical and clinical trials.

## Methods

### Cell lines and primary AML cells

The human AML cell lines MOLM-13 and HL60, and the human osteosarcoma cell line SAOS-2 and the human lung cancer cell line H1299 were purchased from ATCC (American Type Culture Collection, Manassas, VA, USA), while the human AML cell line OCI-AML3 was purchased from DSMZ (Deutsche Sammlung von Mikroorganismen und Zellkulturen GmbH, Braunschweig, Germany). Cell lines were cultured according to manufacturer’s procedure. For patient material, all studies were performed in accordance with the Helsinki declaration and approved by the regional Ethics Committee (REK Vest; http://helseforskning.etikkom.no, Norwegian Ministry of Education and Research). Samples were collected after informed consent, and mononuclear cells were isolated and stored frozen in liquid N_2_ as previously described [60]. Normal peripheral blood lymphocytes were obtained from healthy blood donors (Blodbanken, Haukeland University Hospital, Bergen, Norway). Primary AML cells and normal peripheral blood lymphocytes were cultured in StemSpan SFEM™ (StemCell Technologies Inc., Vancouver, BC, USA).

### Compounds

Nutlin-3 (Cayman Chemical Company, Michigan, USA) and geldanamycin (Sigma-Aldrich, Inc., St Louis, MO, USA) were dissolved in DMSO, and stored at −80°C. When used in cell culture work, the final concentration of DMSO did not exceed 0.1%.

### Western blotting

Western blotting was performed as previously described [22]. The following antibodies were used; p53 (Bp53-12), Mdm2 (SMP-14) (Santa Cruz Biotechnology, CA, USA), Mdm2 (2A10), Mdm2 (IF2), anti-Hsp27 (G3.1) (Calbiochem, San Diego, CA, USA), p21 (SX118) (BD Biosciences, San Jose, CA, USA), phospho-p53 (Ser15), phospho-p53 (Ser20), ac-p53 (Lys382) (Cell Signaling Technologies, Beverly, MA, USA), anti-Histone H2B, anti-Hsp90 (Millipore, Temecula, CA, USA), anti-acetyl-Histone H2B (Lys120) (Upstate cell signaling solutions, Lake Placid, NY, USA), anti-acetyl-Hsp90 (Lys294) (Rockland Immunochemicals, Inc., Gilbertsville, PA, USA), secondary horse radish peroxidase conjugated mouse and rabbit antibody (Jackson ImmunoResearch, West Grove, PA, USA), actin (AC-15) (Abcam plc, Cambridge, UK). Bands were quantified using region of interest analysis on Kodak Molecular Imaging Software version 5.0.1 (Carestream Health, Rochester, NY, USA). Fold induction are given in arbitrary units and are defined as protein of interest/actin following normalization of control.

### Flow cytometry

Flow cytometric analysis was performed as previously described [22], using antibodies against Hsp90 α/β (F-8) PE (Santa Cruz Biotechnology, CA, USA) and Hsp27 (G3.1) PE (Enzo Life Sciences, Farmingdale, NY, USA).

### Plasmids and transfections

p53 cDNA constructs of p53 FL and p53 6KR were previously described [35]. Transfections were performed using X-tremeGENE 9 DNA Transfection Reagent (Roche Diagnostics, GmbH, Mannheim, Germany) according to the manufacturer’s procedure as previously described [61].

### Cell viability and proliferation assays

Evaluation of apoptosis, viability and proliferation in cell lines and primary AML cells after drug treatment was accomplished using Hoechst 33342 (Invitrogen, Carlsbad, Ca, USA), the viability/proliferation reagent WST-1 (Roche Diagnostics GmbH, Mannheim, Germany), ^3^H-thymidine (Amersham International, Amersham, UK) incorporation assay, APOTEST-FITC kit (Nexins Research, Kattendijke, The Netherlands) or Alexa Fluor 488 Annexin V/ Dead Cell Apoptosis Kit (Molecular Probes, Invitrogen, Eugene, Oregon, USA) as previously described [22].

### Immunoprecipitation

Approximately 50 million cells were lysed in Triton® X-100 lysis buffer containing 150 mM NaCl, 50 mM Tris HCl pH 8.0, 1% Triton® X-100 (Plus one, Pharmacia Biotech, Uppsala, Sweden), Complete mini Protease inhibitor cocktail tablet (Roche Diagnostics GmbH, Mannheim, Germany), 5 mM NaF, 1 mM Na-orthovanadate, 10 mM nicotinamide and 1 μM TSA, and immunoprecipitation was carried out using μMACS ProteinG Microbeads (Miltenyi Biotec, Gladbach, Germany) according to the manufacturer’s procedure. The cell lysate was pre-cleared with μMACS Protein G MicroBeads to remove unspecific binding to the beads followed by a pre-clear using an unspecific antibody (Chromatographically purified Rabbit IgG, Invitrogen, Camarillo, CA, USA) and μMACS Protein G MicroBeads to remove unspecific binding to the immunoglobulines, before new μMACS Protein G MicroBeads and anti-acetyl-lysine antibody (4G12) (Millipore, Billerica, MA, USA) were added to the pre-cleared lysate for immunoprecipitation of acetylated proteins. Proteins were eluted in 95°C SDS loading buffer and loaded directly on to a gel for electrophoresis.

### Stable isotope labeling with amino acids in cell culture (SILAC), mass spectrometry and analysis of mass spectrometry data

MOLM-13 cells were grown in SILAC RPMI media with 10% dialyzed FBS, 1% penicillin, 0.1 mg/ml L-Lysine-2HCL and 0.1 mg/ml mg L-Arginine-HCl, or 0.1 mg/ml ^13^C_6_ L-Lysine-2HCl and 0.1 mg/ml mg ^13^C_6_^15^N_4_ L-Arginine-HCl (Pierce SILAC Protein Quantification Kit – RPMI 1640, Thermo Scientific, Pierce Protein Research Products, Rockford, IL, USA) for six passages [25], and incorporation efficiency was determined by mass spectrometric analysis. Cell lysates were mixed at a ratio of 1:1 (5 mg protein of each sample) before immunoprecipitation procedures were performed. Eluted proteins from the immunoprecipitation were separated by one-dimensional gel electrophoresis and stained with Coomassie Blue (Pharmacia Biotech, Uppsala, Sweden). The gel was sliced into 13 gel pieces prior to reduction, alkylation, trypsin digestion and analysis by nano-LC (Ultimate 3000, Dionex, Sunnyvale, CA, USA) coupled to an ESI-Orbitrap (LTQ Orbitrap XL, Thermo Scientific, Bremen, Germany) mass spectrometer as previously described [62]. The peptides were identified and quantified using the MaxQuant and Perseus software (version 1.2.2.5 and 1.2.0.16) [63] with the following settings: carbamidomethyl (C) as fixed modification, and oxidation (M), acetylation (K) and acetylation (protein N-term) as variable modifications. FDR was 1%, MS tolerance was 10 ppm and MS/MS tolerance was 0.7 Da. Only proteins with more than 1 peptide were included in the analysis. All ratios are given as normalized values and are tested with Benjamini-Hochberg FDR test (*p* < 0.05) using significance B.

### Analysis of intracellular levels of heat shock proteins

Intracellular levels of heat shock proteins Hsp27 (phospho-Ser82), Hsp27 (phospho-Ser15), Hsp40, Hsp60, Hsp70 and Hsp90α were determined using the Hsp/Chaperone 8-plex MultiBead kit (Assay Designs, Inc., Ann Arbor, MI, USA) according to manufacturer’s instructions as previously described [17].

### Statistical analysis

In cell viability and proliferation assays, triplicates were analyzed for each sample, and results given as means +/− standard error of mean. Statistical significance of differences in averages was determined using a two-tailed Student’s *t*-test. For statistical comparison between different patient groups, we used Mann–Whitney *U*-test. Correlation analysis was performed using Pearson’s correlation, and synergism was calculated by Bliss Independence analysis. For all statistical analysis, *p <* 0.05 was considered significant. Graphs and calculations were obtained using GraphPad Prism® 5.0 (GraphPad Software, La Jolla, CA, USA). Results from flow cytometric analysis were visualized using TMEV microarray software suite version 4.3.01 (Dana-Farber Cancer Institute, Boston, MA, USA).

## Abbreviations

AML: Acute myeloid leukemia; E2F-1: E2F transcription factor 1; FLT3: FMS-like tyrosine kinase 3; H2B: Histone H2B; HDAC: Histone deacetylase; HSP: Heat shock protein; IP: Immunoprecipitation; LC-MS/MS: Liquid chromatography-tandem mass spectrometry; MDM2: Mouse double minute 2; MDMX: Mouse double minute X; MFI: Median fluorescence intensity; NPM: Nucleophosmin; PCAF: P300/CBP-associated factor; SILAC: Stable isotope labeling with amino acids in cell culture; TP53: Tumor protein p53.

## Competing interests

The authors have no competing interests.

## Authors’ contributions

IH, FSB, SL, ØB and BTG designed the study. IH, HR, HKF, and RH performed experiments. IH, JAO, HR and BT analyzed data. SL contributed with reagents. IH, JAO, BT and BTG wrote the manuscript. EMC critically revised the manuscript. All authors revised the manuscript. All authors read and approved the final manuscript.

## Supplementary Material

Additional file 1: Table S1 text An explanation of the data shown in Table S1 (Additional file 2). Click here for file

Additional file 2: Table S1 A complete list of proteins identified using MaxQuant (version 1.2.2.5) from experiments using anti-acetyl-lysine immunoprecipitation and SILAC (stable isotope labeling with amino acids in cell culture) analysis of MOLM-13 cells treated with nutlin-3. Click here for file

Additional file 3: Table S2 AML patient characteristics of primary AML cells used in the study. Click here for file

Additional file 4: Figure S1 Intracellular heat shock protein levels and sensitivity to nutlin-3 in primary AML cells with wild type TP53. Click here for file
