# Supplementary material for: Molecular mechanisms of nutlin-3 involve acetylation of p53, histones and heat shock proteins in acute myeloid leukemia
Source: Mol Cancer. 2014 May 21;13:116. doi: 10.1186/1476-4598-13-116 (PMC4032636; doi:10.1186/1476-4598-13-116)
Supplement: Additional file 1: Table S1 text — An explanation of the data shown in Table S1 (Additional file 2). [file 1476-4598-13-116-S1.pdf]

**Supplementary Table 1:** A complete list of proteins identified with two or more peptides using MaxQuant (version 1.2.2.5) from experiments using anti-acetyl-lysine immunoprecipitation and SILAC (stable isotope labeling with amino acids in cell culture) analysis of MOLM-13 cells treated with nutlin-3 (6 μM, 6h) is provided. MOLM-13 cells treated with DMSO (control) were labeled with light isotopes of amino acids (L), and nutlin-treated cells were labeled with heavy isotopes of amino acids (H); the reported regulation of proteins in response to nutlin-3 is the **normalized H/L ratio**, given as fold induction of control. Protein identities are sorted according to their normalized H/L ratios. Proteins that were either two-fold upregulated (normalized H/L ratio > 2.0) or downregulated (normalized H/L ratio < 0.5) were considered statistically significant. Table headings for other important columns: **Leading protein:** the accession number for the protein with most matching peptides; **Protein ID:** Lists the IDs for all proteins in this protein group with the leading protein first; **Protein name**: Lists the names of the proteins in this protein group, with the leading protein first; **Peptides:** Represents the number of identified peptides for the protein group; **Unique peptides:** Represents the number of unique peptides for the leading protein. A more detailed explanation of the column headers is available via the MaxQuant website: http://maxquant.org.
