# Supplementary material for: Molecular mechanisms of nutlin-3 involve acetylation of p53, histones and heat shock proteins in acute myeloid leukemia
Source: Mol Cancer. 2014 May 21;13:116. doi: 10.1186/1476-4598-13-116 (PMC4032636; doi:10.1186/1476-4598-13-116)
Supplement: Additional file 3: Table S2 — AML patient characteristics of primary AML cells used in the study. [file 1476-4598-13-116-S3.pdf]

Supplementary Table 2: AML patient characteristics.

Patient Number	Age (Yrs)/Sex	Previous Malignancy	AML FAB	Immunophenotype (% +ve Cells) CD13	CD14	CD15	CD33	CD34	Karyotype	Cytogenetics and Mutational Status FLT3	NPM1	TP53	Disease Res.	Survival (Months)
1	32/F		M5	82	1	27	100	10	del 5	Wt	Mut	Wt	No	> 30
2	72/M	MDS	M1	84	1	10	99	57	Multiple	ITD	Wt	ND	NA	14
3	77/F		M1	38	1	2	96	1	ND	ND	ND	Wt	NA	0
4	46/F	Relapse	M1	ND	ND	ND	ND	ND	Normal	Wt	Mut	Wt	No	20.25
5	62/M		M1	ND	ND	ND	Pos	Pos	Normal	Wt	Wt	Wt	No	NA
6	47/M		M4	70	1	1	30	100	t(8;21)	Wt	ND	Wt	No	> 7
7	64/F		M2	47	1	21	84	3	Normal	ITD	Mut	ND	Yes	12
8	75/F		M1	43	11	2	55	23	ND	ITD	Wt	Wt	NA	NA
9	70/F		ND	78	1	36	98	1	Normal	Wt	Mut	Wt	NA	2
10	29/M	Relapse	M4	92	5	24	96	55	Normal	ITD+Asp835	Mut	Wt	Yes	3
11	58/M		M5	ND	ND	ND	70	82	Normal	Wt	Wt	Wt	No	NA
12	59/F		M4	41	10	2	74	2	Normal	ITD	Mut	Wt	Yes	> 9
13	53/M		M0	56	ND	46	46	59	+13	Wt	Wt	Wt	No	NA
14	74/F		M4	41	3	8	64	30	ND	ITD	Wt	Wt	NA	2
15	79/M		M5	46	45	78	89	32	ND	ND	ND	Wt	NA	NA
16	61/M	Relapse	M2	80	2	26	98	99	45, XY, -7	Wt	Mut	Wt	Yes	18
17	77/F		M1	72	1	66	3	87	ND	ITD	Wt	Wt	NA	8
18	48/M		M4c	97	2	48	75	78	46XY, inv(16)	Wt	Wt	Wt	No	> 36
19	45/M		M4	90	1	24	98	1	Normal	Wt	Mut	Wt	No	> 15
20	63/M		M1	82	32	ND	41	68	Normal	Wt	ND	Wt	Yes	7
21	72/M		M5	31	40	69	99	1	Normal	Wt	Mut	Wt	Yes	1.25
22	63/F		M4	61	1	26	100	51	Normal	ITD	Wt	Wt	Yes	6
23	59/F		M2	97	1	4	66	99	-7	Wt	Wt	ND	Yes	3
24	67/M		M0	6	1	8	9	99	del (5)	ITD	Wt	ND	No	NA
25	68/F	Relapse	M1	100	1	35	28	98	Normal	ITD	Wt	ND	NA	2
26	49/F	Relapse	M2	97	1	1	84	85	Multiple	ND	ND	Wt	NA	7.5
27	75/F		M4	98	9	55	75	76	Normal	ITD	Wt	Wt	Yes	5
28	42/F		M5	58	90	95	100	1	Normal	Wt	Mut	Wt	No	> 30
29	69/M		M0	64	12	14	62	65	ND	ITD	ND	Wt	Yes	NA
30	82/M	PVR	M0	66	1	6	26	60	ND	ND	ND	Wt	NA	0.5
31	29/F		M5	40	20	4	ND	79	Normal	ITD+Asp835	Wt	Wt	Yes	> 10
32	80/F		M2	98	3	14	87	96	Multiple	Wt	Wt	Mut (P151T, Hetero)	Yes	0.5
33	63/M	MDS	M1	1	1	1	70	100	Multiple	Wt	Wt	Mut (T376A, Hetero)	NA	2
34	67/M	Relapse	M1	ND	ND	ND	ND	ND	Normal	ITD	Wt	ND	NA	1
35	59/F		M4	63	5	8	99	4	Normal	ITD	Mut	Wt	No	9
36	81/F	MDS	M1	58	1	3	61	73	Normal	Wt	Wt	Wt	NA	1
37	64/F	Relapse	M4	60	6	21	58	26	42-46,XX,-16,-22	Wt	Wt	Mut (Del 193-1225, Hetero)	NA	2
38	48/F		M1	99	1	5	67	35	Normal	ITD	Mut	Wt	No	NA
39	36/M		M5	92	14	43	97	84	Inv(16), +8 ,+22	ITD	Wt	Wt	No	> 12
40	33/M		M1	98	1	1	96	99	Normal	Wt	Wt	Wt	No	> 34

Patient Number	Age (Yrs)/Sex	Previous Malignancy	AML FAB	Immunophenotype (% +ve Cells) CD13	CD14	CD15	CD33	CD34	Cytogenetics and Mutational Status Karyotype	FLT3	NPM1	TP53	Disease Res.	Survival (Months)
41	69/M		M4	70	19	31	52	74	ND	ITD	Wt	Wt	NA	NA
42	61/F		M5	43	91	82	99	1	Normal	ITD	Wt	Wt	Yes	5
43	72/F	Myelofibrosis	M1	10	1	7	47	90	ND	ND	ND	Wt	NA	2
44	67/F		M4	80	3	38	95	79	t(16;16),+22	Wt	Wt	Wt	NA	NA
45	68/M		M2	100	1	54	30	76	Multiple	Wt	Wt	Wt	NA	NA

CD; cluster of differentiation, del; Deletion, F; Female, FAB; French-American-British, FLT3; FMS-like tyrosine kinase 3, Hetero; Heterozygot, inv; Inversion, ITD; Internal tandem duplication, M; Male, MDS; Myelodysplastic syndrome, Mut; Mutated, NA; Not available, ND; Not done, NPM1; Nucleophosmin 1, PVR; Polycythema vera, Res; Resistance, Wt; Wild-type, Yrs; Years of age, +ve; Positive
