# Supplementary material for: Molecular mechanisms of nutlin-3 involve acetylation of p53, histones and heat shock proteins in acute myeloid leukemia
Source: Mol Cancer. 2014 May 21;13:116. doi: 10.1186/1476-4598-13-116 (PMC4032636; doi:10.1186/1476-4598-13-116)
Supplement: Additional file 4: Figure S1 — Intracellular heat shock protein levels and sensitivity to nutlin-3 in primary AML cells with wild type TP53. [file 1476-4598-13-116-S4.pdf]

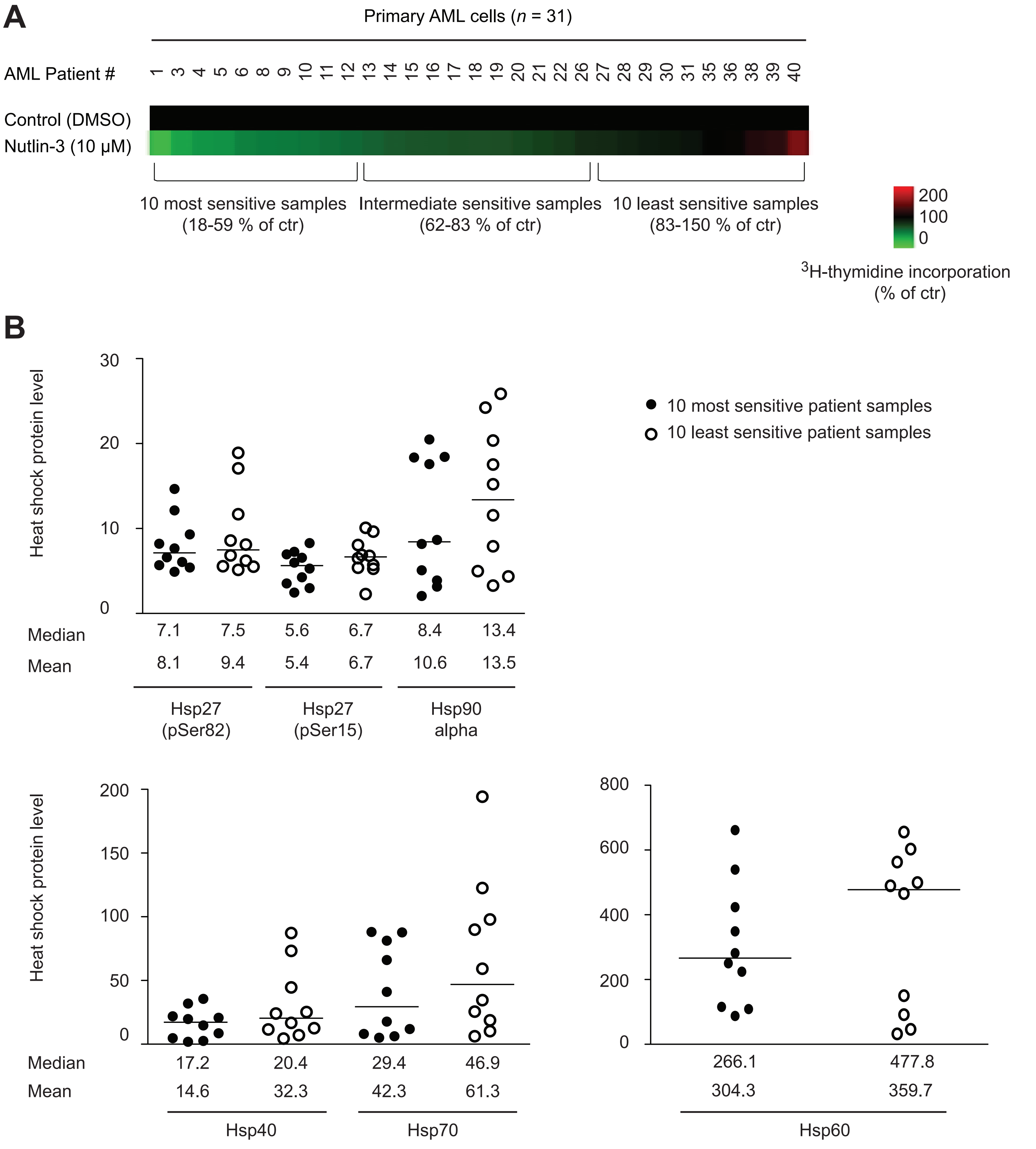

Supplementary Figure 1

Supplementary Figure 1. Intracellular heat shock protein levels and sensitivity to nutlin-3 in primary AML cells with wild type TP53. (A) Sensitivity to nutlin-3 (10 μM, 24 hours) in 31 primary AML samples with wild type TP53 was determined by ^{3}H-thymidine incorporation assay, and samples were analyzed in triplicates. Intracellular levels of heat shock proteins Hsp27 (phospho-Ser82), Hsp27 (phospho-Ser15), Hsp40, Hsp60, Hsp70 and Hsp90α for all samples were determined using Hsp/Chaperone 8-plex MultiBead kit and flow cytometric analysis. Samples were analyzed in duplicates. **(B)** Median values of heat shock protein levels were determined for patient samples that were sensitive (10 most sensitive ranging from 18-59% viability of control) and non-sensitive (10 least sensitive ranging from 83-above 100% viability of control) to nutlin-3, and are shown in the figure together with values for individual patient samples. Mean values are given below. For Hsp27 (phospho-Ser82) and Hsp27 (phospho-Ser15) levels are given as u/ml; for Hsp40, Hsp60, Hsp70 and Hsp90α levels are given as ng/ml.
